# Curcumin inhibition of JNKs prevents dopaminergic neuronal loss in a mouse model of Parkinson’s disease through suppressing mitochondria dysfunction

**DOI:** 10.1186/2047-9158-1-16

**Published:** 2012-08-20

**Authors:** Jing Pan, Hui Li, Jian-Fang Ma, Yu-Yan Tan, Qin Xiao, Jian-Qing Ding, Sheng-Di Chen

**Affiliations:** 1 Department of Neurology and Neuroscience Institute, Ruijin Hospital, Shanghai Jiao-Tong University School of Medicine, Shanghai, 200025, P.R. China; 2 Institute of Health Science, Shanghai Institutes for Biological Sciences (SIBS), Chinese Academy of Sciences (CAS) & Shanghai Jiao-Tong University School of Medicine, Shanghai, 200025, P.R. China

## Abstract

Curcumin,a natural polyphenol obtained from turmeric,has been implicated to be neuroprotective in a variety of neurodegenerative disorders although the mechanism remains poorly understood. The results of our recent experiments indicated that curcumin could protect dopaminergic neurons from apoptosis in a 1-methyl-4-phenyl-1,2,3,6-tetrahydropyridine (MPTP) mouse model of Parkinson’s disease (PD). The death of dopaminergic neurons and the loss of dopaminergic axon in the striatum were significantly suppressed by curcumin in MPTP mouse model. Further studies showed that curcumin inhibited JNKs hyperphosphorylation induced by MPTP treatment. JNKs phosphorylation can cause translocation of Bax to mitochondria and the release of cytochrome c which both ultimately contribute to mitochondria-mediated apoptosis. These pro-apoptosis effect can be diminished by curcumin. Our experiments demonstrated that curcumin can prevent nigrostriatal degeneration by inhibiting the dysfunction of mitochondrial through suppressing hyperphosphorylation of JNKs induced by MPTP. Our results suggested that JNKs/mitochondria pathway may be a novel target in the treatment of PD patients.

## Introduction

Parkinson’s disease (PD) is second only to Alzheimer’s disease (AD) as the most common and debilitating age-associated human neurodegenerative disorder. A host of environmental, genetic, and immune cues have been associated with the onset of this disease [1]. Clinical symptoms of PD include tremor, bradykinesia, rigidity, and postural instability [2,3]. Pathologically, it is characterized by gliosis and progressive degeneration of the dopaminergic neurons associated with the presence of intracytoplasmic inclusions (Lewy bodies) in the substantia nigra pars compacta (SNc) [2,3]. The symptoms of PD can be alleviated by drugs that enhance dopamine function, among which L-dopa is considered the most effective one. However, L-dopa fails to halt the progression of PD. Aside from having undesirable side effects, such as motor fluctuations and dyskinesias, the therapeutic effect of L-dopa diminishes after about two years of treatment [4]. Moreover, long term use of L-dopa may actually damage neurons, accelerating neuronal apoptosis. Since programmed cell death plays a key role in the neurodegenerative processes in PD [5], new generation of neuroprotective agents against apoptosis may improve the prognosis of PD.

Curcumin has been implicated to be neuroprotective in a variety of neurodegenerative disorders such as AD and cerebral ischemi [6,7]. Epidemiological evidence from India has related the huge consumption of turmeric (curcumin is its essential component) to its lowest prevalence rates of AD and PD in the world [8]. As a matter of fact, curcumin is now in Phase II clinical trials for AD [9]. Curcumin has been reported to be a good inhibitor of c-Jun N-terminal kinase (JNK) mediated gene transcription [10]. JNK is a important member of mitogen-activated protein kinases (MAPK) family, which can be activated by a variety of stimuli including neurotoxic insults, environmental stress and apoptotic agents [11-13]. JNK is composed of three different isoforms, JNK1, JNK2 and JNK3. In contrast to JNK1 and JNK2, which are ubiquitously expressed, JNK3 is largely restricted to the brain and is most consistently associated with neuronal death [14] Our previous studies and others suggested that JNK plays an important role in mediating MPTP-induced neurotoxicity. CEP1347, a specific JNK pathway inhibitor, attenuates the loss of nigrostriatal dopaminergic neurons after the exposure to MPTP [15] SP600125 (a selective inhibitor of JNK) prevents dopaminergic neurons from death and decreases the loss of catecholamines in the striatum [16] by partially inhibiting JNK pathway. Therefore, it is reasonable to assume that blockade of JNK pathway may prevent or effectively slow down the progression of PD. Nevertheless, an understanding of the molecular mechanisms by which JNK regulates apoptosis should provide insights into the treatment of PD.

Previous studies demonstrated that JNK can promote cell death by regulating the activation of substrates, such as Bcl-2 family members [17]. The Bcl-2/Bax heterodimer is the active component for death protection [18,19]. Phosphorylation of Bcl-2 may possibly release Bax from Bcl-2/Bax dimmers [20-22]. The preapototic protein Bax forms pores in the outer mitochondrial membrane to release cytochrome c [23], thus promoting apoptosis,. On death induction, cytochrome c not only translocates into the cytosol, but furthermore can be abundantly detected in the extracellular medium. Thus, release of cytochrome c is considered as an indication of mitochondrial dysfunction [24]. It is therefore possible that through regulating the activation of some Bcl-2 family members, activated JNK pathway increase mitochondrial membrane permeability and the subsequent release of apoptogenic factors, which could ultimately contribute to mitochondria mediated apoptosis.

Whether curcumin could inhibit the abnormal activation of JNK induced by MPTP, thus prevent the triggering of a series downstream effects that lead to apoptosis is unknown. In this study, the inhibitory effect of curcumin to the MPTP-induced activation of JNK was evaluated. Using a MPTP-induced PD mice model, we demonstrated that curcumin could suppress the activation of the JNK in PD mice induced by MPTP. Furthermore we demonstrated that curcumin could decrease MPTP-induced injuries to dopaminergic cell bodies and terminals via inhibiting mitochondria mediated apoptosis.

## Materials and methods

### Animals

Studies were conducted in male C57BL/6 mice (8 ~ 10 weeks old, weighing 19 ~ 22 g). Five animals were housed per cage in a temperature-controlled (25°C) room under a 12:12-h light:dark cycle with ad libitum access to food and water for 1 week before the experiment. The mice were injected intraperitoneally (i.p.) 5 times (for five consecutive days) with 30 mg/kg/day MPTP-HCl (Sigma, St. Louis, MO) or a corresponding volume of saline alone. One day after the last MPTP injection, the curcumin treated mice groups were treated with an intraperitoneal injection of curcumin (sigma) at 50 mg/kg/day for five consecutive days. Equal dose of DMSO instead of curcumin were given to the control group. At the indicated time points, the animals were killed, and their brains were processed for further analysis.

### Immunohistochemistry

Mice were perfusion-fixed on Day 10 with 4% paraformaldehyde in 0.1 M sodium phosphate buffer (pH 7.4). Brains were removed quickly and further fixed with the same fixation solution at 4°C overnight. Post-fixed brains were embedded by paraffin, followed by preparation of coronal sections using a microtome. The paraffin-embedded brain sections were deparaffinized with xylene and rehydrated by ethanol at graded concentrations of 100-70% (v/v), followed by washing with water.

Immunoreactivity was determined by the avidin–biotin–peroxidase method. Briefly, sections were deparaffinized with xylene and rehydrated by ethanol at graded concentrations and distilled water. High-temperature antigen retrieval was performed in 1 mM citrate buffer for 15 min. To block endogenous peroxidase activity, sections were incubated for 30 min in 1% H_2_O_2._ After being blocked with 5%(v/v) normal goat serum in PBS for 1 h at 37°C, sections were incubated with a mouse polyclonal antibody against tyrosine hydroxylase (TH, 1:8000) at 4°C for 24 hours. These sections were then incubated with biotinylated goat-anti-mouse secondary antibody overnight and subsequently with avidin–conjugated horseradish peroxidase for 1 h at 37°C. Finally, sections were incubated with peroxidase substrate diaminobenzidine (DAB) until desired stain intensity developed [25].

### Electron Microscopy

Mitochondria isolated from ischemic and reperfused myocardial tissue in several kinds of buffer by density centrifugation was resuspended and fixed with 2% glutaraldehyde in 0.1 M PBS buffer. Mitochondria were post-fixed utilizing 1% OsO4. En bloc staining with uranyl acctate was followed by dehydration and embedding. Embedded samples were sectioned and affixed to grids according to standard protocols. Mitochondrial ultrastructure was then evaluated by transmission microscopy.

### Immunoprecipitation and immunoblotting

Tissue homogenates (400 μg of protein) were diluted four-fold with 50 mM HEPES buffer (pH 7.4), containing 10% glycerol, 150 mM NaCl, 1% Triton X-100, 0.5% NP-40, and 1 mM each of EDTA, EGTA, PMSF and Na_3_VO_4_. Samples were preincubated for 1 h with 20 μl protein A sepharose CL-4B (Amersham, Uppsala, Sweden) at 4°C, and then centrifuged to remove proteins adhered non-specifically to protein A. The supernatants were incubated with 1-2 μg primary antibodies for 4 h or overnight at 4°C. Protein A was added to the tube for another 2 h incubation. Samples were centrifuged at 10,000 × g for 2 min at 4°C and the pellets were washed with HEPES buffer for three times. Bound proteins were eluted by boiling at 100°C for 5 min in SDS-PAGE loading buffer and then isolated by centrifugation. The supernatants were used for immunoblotting analysis. Proteins were separated on polyacrylamide gels and then electrotransferred onto a nitrocellulose membrane (Amersham, Buckinghamshire, UK). After being blocked for 3 h in Tris-buffered saline with 0.1% Tween-20 (TBST) and 3% bovine serum albumin (BSA), membranes were incubated overnight at 4°C with primary antibodies in TBST containing 3% BSA. After washing for 30 min in TBS with gentle agitation, the membrane was incubated with horseradish peroxidase-conjugated anti-mouse/rabbit IgG secondary antibody at room temperature for 2 h ([26-28]). Signals were developed using ECL Western Blotting Detection kit (Amersham-Pharmacia Biotech, Little Chalfont, UK). Band intensities were quantified by densitometric analyses using an AxioCam digital camera (ZEISS, CTED PROOF Germany) and a KS400 photo analysis system (Version 3.0).

### Statistical evaluation

Values were expressed as mean S.D. and obtained from at least six independent experiemnts. Statistical analysis of the results was carried out by Student’s t-test or one-way analysis of the variance (ANOVA) followed by the Duncan’s new multiple range method or Newman-Keuls test. P-values less than 0.05 were considered significant.

## Results

### Effects of curcumin on MPTP-induced loss of dopaminergic neurons

To investigate whether treatment of curcumin would have neuroprotection against MPTP-induced dopaminergic neuronal death, C57BL/6 mice were subjected MPTP lesion. Mice were treated with curcumin or DMSO by injection for 5 consecutive days after last MPTP injection.

**Figure 1:**
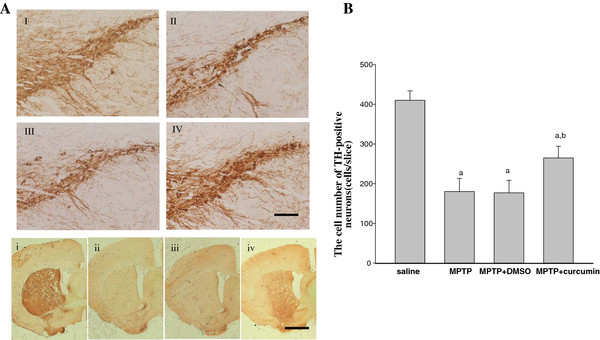
**Effects of curcumin on cell death and survival in SNc.** Curcumin attenuated MPTP-induced DA terminal loss in striatum and dopaminergic neuronal death in the SNc. TH-stained sections of SNc in saline group (I), mice subjected MPTP-induced lesion (II), administration of the curcumin (III) and DMSO (IV) following MPTP injection. When compared to Curcumin treated mice (iv), DMSO-treated mice following MPTP lesion showed marked reduction in TH positive fibres in striatum (iii). (i, ii) TH staining in striatum of saline and MPTP-treated mice. Scale bars: (I,II,III,IV) =400 μm; (i,ii,iii,iv) =100 μm. (**B**) Quantitative analysis of the protective effects of curcumin against MPTP-induced mice model of nigrostriatal damage. ^*a*^*P* < 0.05 vs. saline control ^*b*^*P* <0.05 vs. MPTP injection groups.

We first examined the effect of curcumin on tyrosine hydroxylase (TH)-positive neurons in SNc of MPTP animal model. As shown in Figure 1A (I), MPTP induced marked nigral cell death (Figure 1A(II)). However, administration of curcumin clearly rescued the neurodegeneration caused by MPTP (Figure 1A (IV)). At the same time, as the control, DMSO did not show any protection (Figure 1A (III)). The results indicated that curcumin was capable of protecting neurons against MPTP-induced injury. TH immunostaining in the striatum was assessed as an indication of dopaminergic axon. The results revealed curcumin treatment minimized the decreased densities of dopaminergic axon in the caudate-putamen (CP) region of the striatum (Figure 1A (iv)), while the DMSO did not have such effect (Figure 1A (iii)).

### Electron micrographs analysis of morphology of mitochondria isolated

**Figure 2:**
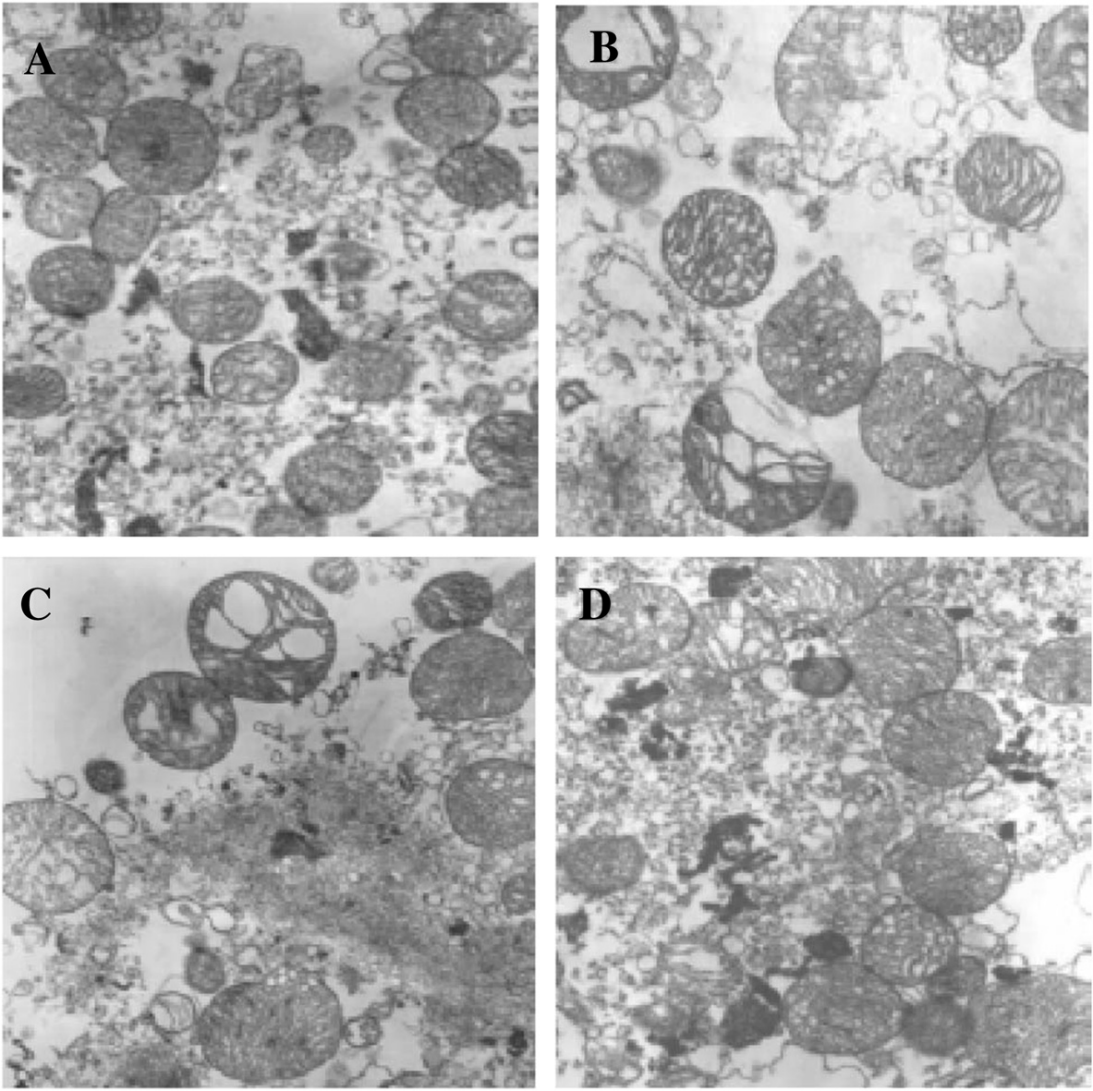
**Electron micrographs analysis of morphology of mitochondria isolated.** Structural analysis of mitochondria isolated from saline group (**A**), mice subjected MPTP-induced lesion (**B**), administration of the curcumin (**D**) and DMSO (**C**) following MPTP injection. Following isolation,mitochondria were processed for electron microscopic analysis as described in Materials and Methods. Electron micrographs are at a magnification of 10,000X (n = 2).

With mitochondrial dysfunction being thought to be related to cell death [29]. We tested whether morphology of mitochondria isolated from correlation is in accordance with curcumin treatment significantly reducing infarct size. Electron microscopy reveals basically formed membranes and clearly descernable cristae in the mitochondria isolated from saline and curcumin treatment mouse. Whereas the mitochondria isolated from control groups were severely swollen, with partially disrupted outer membrane and fragmentation of the cristae. Furthermore, mitochondria in curcumin group appear to morphologically be better than that from other groups(Figure 2).

### Effects of curcumin on MPTP-treated activation and expression of JNKs

JNK signaling is an important contributor to MPTP-induced apoptosis, but JNK and its isoforms (JNK1, JNK2, and JNK3) have distinct roles. As aforementioned, targeted disruption of the *Jnk3* gene not only reduced the downstream effector c-Jun phosphorylation, but also remarkably protected mice from brain injury after MPTP lesion. These studies indicate the functional diversity of JNK isoforms and suggest that JNK3 is a critical component of MPTP-induced JNK signaling and neuronal apoptosis.

**Figure 3:**
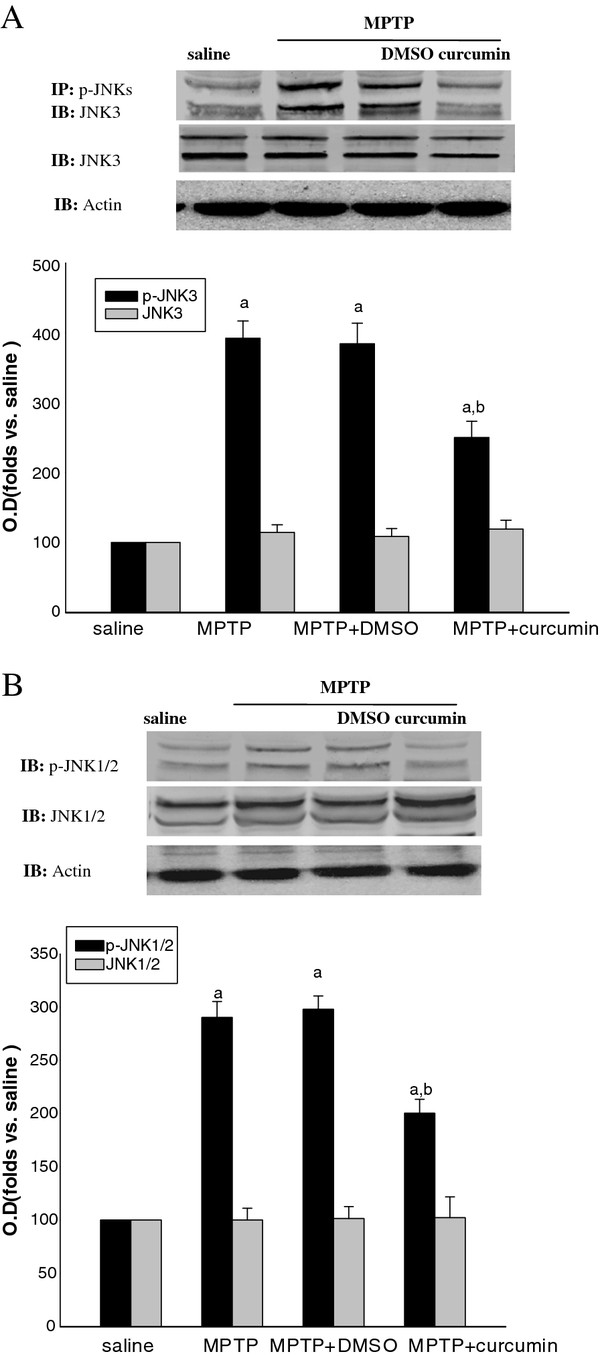
**Effects of curcumin on the phosphorylation of JNKs.** Effects of curcumin on the phosphorylation of JNKs in SNc. Bands corresponding to JNK3 and p-JNK3 were scanned and the intensities were represented as folds vs. saline animals. Phosphorylation of JNK3 was examined by immunoprecipitation (IP) with anti-p-JNKs antibody followed by immunoblotting (IB) with antibody against JNK3. P-JNK1/2 was examined by IB with antibody against p-JNK1/2. The protein levels of JNK1/2 and J NK3 were examined by immunoblotting with anti-JNK1/2 and anti-JNK3 antibodies. Data are expressed as the mean ± SD and as folds vs. respective saline groups. ^*a*^*P* < 0.05 vs. saline; ^*b*^*P* <0.05 vs. MPTP groups (n = 6 mice).

To elucidate the effects of curcumin on the activation of JNKs, JNK1/2/3 phosphorylation were investigated in MPTP lesioned animals. As indicated in Figures 3, the results of western blots showed that the phosphorylation and total level of JNK1/2 and JNK3 were significantly increased after MPTP injection. However, as shown in Figure 3, the treatment of curcumin remarkably inhibited the phorsphorylation of JNK3 rather than JNK1 and 2. The same dose of DMSO did not have the same effect. The protein levels of JNKs were not affected by either curcumin or DMSO treatment. Our study demonstrates that treatment of curcumin attenuated the activation of JNKs especially JNK3 induced by MPTP lesion. Moreover, the fact that the level of JNK3 activation was inhibited more than that of JNK1/2 activation *in vivo* indicated that the activation of JNK3 is more important in the mechanism of dopaminergic neuronal death induced by MPTP.

### Curcumin inhibits the phosphorylation of Bcl-2 and the release of Bax from Bcl-2/Bax dimmers

**Figure 4:**
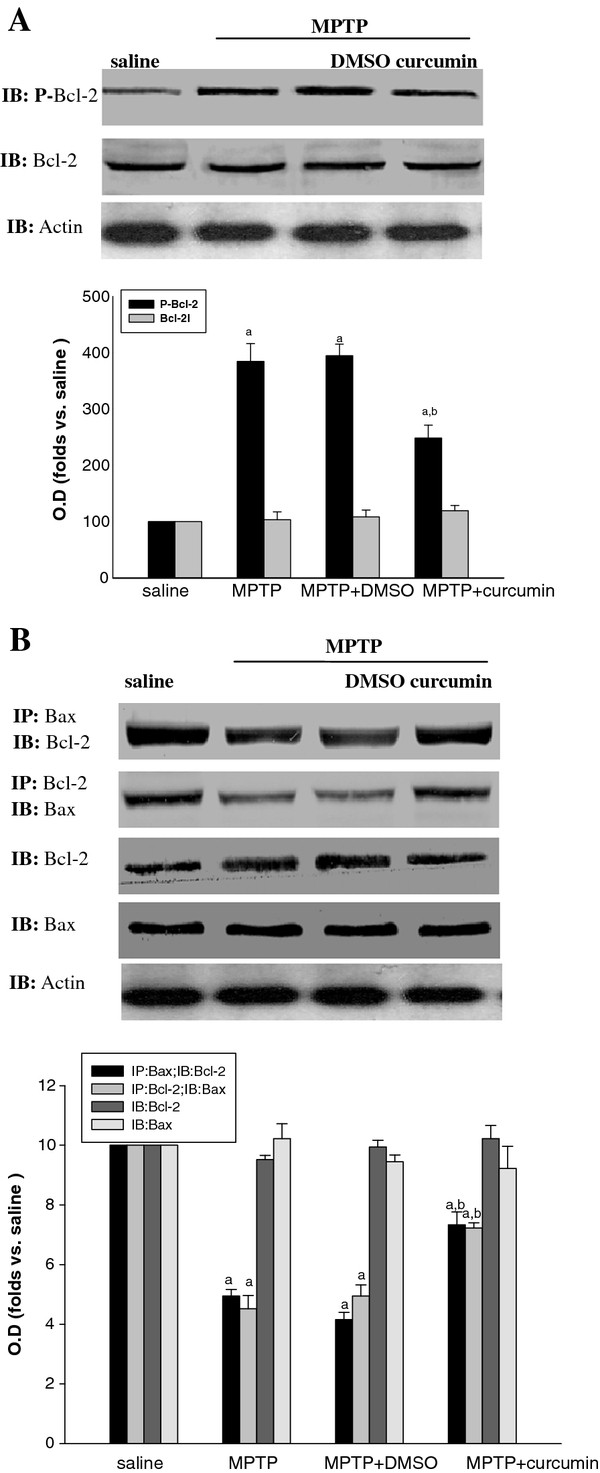
**Effects of curcumin on the altered phosphorylation of Bcl-2 and interactions of Bcl-2 with Bax.** (**A**) Effects of curcumin on the increased phosphorylation of Bcl-2 induced by MPTP in SNc. Western blot probed with antibodies to phosphorylated Bcl-2 (p-Bcl-2, Ser87) and Bcl-2. Bands corresponding to p-Bcl-2 and Bcl-2 were scanned and the intensities were represented as folds vs. saline groups. Data are expressed as mean ± SD (n = 6). ^a^*P* < 0.05 vs. respective saline, ^*b*^*P* < 0.05 vs. MPTP groups. (**B**) Co-immunoprecipitation analysis of interactions between Bcl-2 and Bax. Bands corresponding to Bax and Bcl-2 were scanned and the intensities were represented as folds vs. saline groups. Data are expressed as mean ± SD. ^a^*P* < 0.05 vs. respective saline, ^*b*^*P* < 0.05 vs. MPTP groups. (n = 6 mice).

JNK can promote cell death by regulating the activation of some non-nuclear substrates, such as Bcl-2 family members. Previous studies have also indicated that phosphorylation inactivates Bcl-2, thus promoting apoptosis, possibly by freeing Bax from Bcl-2/Bax dimmers. The Bcl-2/Bax heterodimer is the active component for death protection. Our previous study indicated that K252a (the inhibitor of JNK upsteam) rescued 6-OHDA-induced dopaminergic neuronal death in SNc via suppressing the phosphorylation of Bcl-2 [25]. Since curcumin could inhibit the activation of JNK, we supposed that treatment of curcumin should have the ability to inhibit the phosphorylation of Bcl-2 proteins, increase the interaction of Bcl-2 with Bax. Similar to the result, curcumin inhibited the phosphorylation of Bcl-2 in SNc (Figure 4A and B). In this study, we found that phosphorylated Bcl-2 was not shown to interact with Bax during MPTP lesion (data not shown). Furthermore, our results demonstrated that MPTP decreased interaction of Bcl-2 with Bax and curcumin inhibited the decreased interaction of Bcl-2 with Bax (Figure 4B).

### Curcumin attenuates Bax translocation and the release of cytochrome c

**Figure 5:**
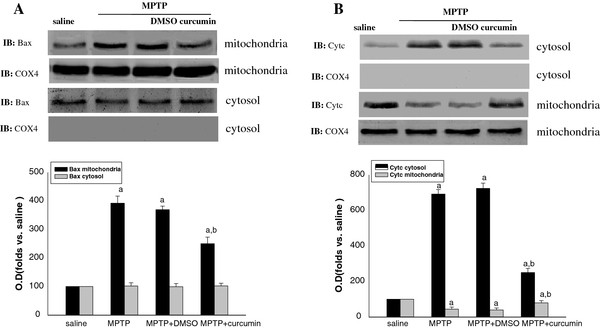
**Curcumin attenuated the mitochondrial apoptosis-signaling pathway in SNc.** (**A**) Effects of curcumin on the level of Bax induced by MPTP in cytosol and mitochondria in SNc. (**B**) Effects of curcumin on the level of cyt c induced by MPTP in cytosol and mitochondria in SNc. COX4 was strongly expressed in the mitochondrial fraction and did not decrease after MPTP lesion, while no immunoreactivity was seen in the cytosolic fraction of SNc region in both the saline and MPTP groups. Bands corresponding to Bax and cyt c were scanned and the intensities were represented as folds vs. saline groups. Data are expressed as the mean ± SD and as folds vs. respective saline. ^*a*^*P* < 0.05 vs. saline; ^*b*^*P* <0.05 vs. MPTP groups (n = 6 mice).

To elucidate the involvement of mitochondria-mediated apoptotic pathway during MPTP lesion and the action of JNK activity on Bax translocation and the release of cytochrome c, level of Bax and cytochrome c in mitochondria and cytosol was examined by Western blotting. We first determined whether Bax translocates from cytosol to mitochondria after lesion. Using Western blotting analysis on different subcellular fraction, we found that the level of Bax was significantly increased in the mitochondria, but the level of Bax was not markedly decreased in cytosol (Figure 5A). We assume that the overwhelming majority of Bax were located in cytosol, thus partial translocation of Bax did not significantly affect the total protein level of Bax in cytosol. Moreover, we examined whether the inhibition of JNKs by curcumin contributes to attenuating Bax translocation. The inhibitory effect of curcumin on Bax translocation in the mitochondrial fraction reached a statistical difference compared with DMSO (Figure 5). A significant amount of mitochondrial cytochrome c was detected in the saline controls and decreased after MPTP lesion, corresponding to a marked increase in the cytosolic fraction (Figure 5). The inhibitory effect of curcumin on the release of cytochrome c in the cytosol fraction reached a statistical difference compared DMSO (Figure 5). To elucidate whether other mitochondrial proteins were released from mitochondria, we determined the cytochrome c oxidase level in the cytosolic and mitochondrial fraction using cytochrome c oxidase subunit IV antibody. The cyt c oxidase subunit IV was detected only in the mitochondrial fraction but not in the cytosolic fraction in saline, MPTP lesion and application of curcumin groups (Figure 5). These results suggested that cytochrome c oxidase was not co-released with cytochrome c from mitochondria.

## Discussion

The most direct evidence for disrupted mitochondrial metabolism has come from studies of autopsy tissue and other tissue samples and *in vitro* cell cultures derived from patients with PD [30]. Mitochondrial dysfunction, due to either environmental or genetic factors, can trigger the apoptotic death of dopaminergic cells in PD. As a matter of fact, results from western blot and electron microscope indicated that cytochrome c releasing and mitochondrial intact were destroyed in MPTP mice. The Bcl-2/Bax heterodimer is the active component for death protection. Previous studies have also indicated that phosphorylation inactivates Bcl-2, thus promoting apoptosis, possibly by freeing Bax from Bcl-2/Bax dimmers [20-22] ,The proapoptotic protein Bax released from Bcl-2/Bax dimmers and act as the channels for either ions or proteins by forming pores in the outer mitochondrial membrane that release cytochrome c [31]. The phosphorylation of Bcl-2 is regulated by JNK [32,33].

A large and growing of evidence suggests that the JNK pathway can function in a pro-apoptotic manner. *In vivo*, the small molecule CEP-1347 inhibits MPTP-mediated JNK signaling at a dose that attenuates MPTP-mediated nigrostriatal dopaminergic loss [15]. In addition, SP600125 also increases striatal catecholamine concentrations, resulting in behavioral changes [16]. As a natual inhibitor of JNK, curcumin, the essential extract from turmeric, is well esablished to exert neuroprotection in animal models of degenerative diseases such as cerebral ischemia, Alzheimer’s disease [9,34,35]. Our previous study and others suggested that JNK3 plays a more important role than JNK1/2 in neuron death and may serve as a potential target for neuroprotective therapies. Ablation of JNKs can not only protect dopaminergic neurons against MPTP-induced neurodegeneration but also improve the motor function in mouse model of PD [36]. As a matter of fact, results from western blot and immunoprecipitation show high fidelity to our hypothesis that curcumin could inhibiteJNKs activation.

Our study also shows that curcumin could attenuate the phosphorylation of Bcl-2 proteins, increase the interaction of Bcl-2 with Bax, and prevent Bax from translocating to sytosol induced by MPTP. Furthermore, curcumin keeps the mitochondria integrity and preserves cytochrome c loss from mitochondria which normally is considered critical role in apoptosis MPTP-intoxicated mice. Since curcumin treatment can inhibit the activation of JNKs mitochondria pathway induced by MPTP lesion, we inferred that application of curcumin would also restore dopaminergic terminals as well as SNc cell bodies from degeneration. Our present study indicated that curcumin actually had the ability to prevent dopaminergic neurons from degeneration following MPTP insult. At the same time, results from TUNEL provided strong evidence that curcumin could protect the dopaminergic neurons from apoptosis. Moreover, curcumin is the major monomer from turmeric extract. Turmeric has been utilized in traditional Indian cuisine and medicine without any known major toxic effect [37]. Thus, the protective effect of curcumin shown here is likely clinically relevant, as significant neuroprotection was achieved when curcumin was administered after the onset of PD. At the same time, we can conclude that selective inhibition of JNKs mitochondria pathway activation may be of therapeutic benefit for PD patients.

## Abbreviations

DA: Dopaminergicrgic; SNc: Substantia nigra pars compacta; MAPK: Mitogen-activated protein kinase; MPTP: 1-methyl-4-phenyl-1,2,3,6-tetrahydropyridine; JNK: c-Jun N-terminal protein kinase; DMSO: Dimethyl sulfoxide; IP: Immunoprecipitation; IB: Immunoblotting; PBS: Phosphate-buffered saline; TH: Tyrosine hydroxylase; 6-OHDA: 6-hydroxydopaminergic; DTT: 1,4-dithiothreitol; BSA: Bovine serum albumin; DAB: Diaminobenzidine; MOPS: 3-(N-morpholino) propanesulfonic acid; TBST: Tris-buffered saline with 0.1% Tween 20; PMSF: Phenylmethylsulfonyl fluoride; SDS–PAGE: Sodium dodecyl sulfate–polyacrylamide gel electrophoresis.

## Competing interests

The authors declare no competing interests.

## Authors’ contributions

JP and HL made substantial contributions to conception and design, acquisition of data, and analysis and involved in drafting the manuscript. J-FM and Y-YT participated in the design of the study and performed the statistical analysis. QX made interpretation of data and involved in revising it critically for important intellectual content. J-QD and S-DC helped critically revise and gave final approval to the manuscript. All authors read and approved the final manuscript.
